# Integration of COVID-19 and TB screening in Kampala, Uganda: healthcare provider perspectives

**DOI:** 10.1186/s43058-023-00391-w

**Published:** 2023-01-17

**Authors:** Fred C. Semitala, Rodgers Katwesigye, Dennis Kalibbala, Mary Mbuliro, Rejani Lalitha, Darius Owachi, Edgar Atine, Josephine Nassazi, Stavia Turyahabwe, Moorine Sekadde

**Affiliations:** 1 Department of Internal Medicine, School of Medicine, Makerere University College of Health Sciences, Kampala, Uganda; 2 Makerere University Joint AIDS Program (MJAP), Kampala, Uganda; 3 Kiruddu National Referral Hospital, Kampala, Uganda; 4 Ministry of Health Uganda, National Tuberculosis and Leprosy Program, Kampala, Uganda

**Keywords:** Tuberculosis, COVID-19, Healthcare providers, Integrated screening

## Abstract

**Background:**

Following the first wave of the COVID-19 outbreak, Uganda experienced a 40% drop in tuberculosis (TB) screening by June 2020. We sought to identify barriers to and facilitators of integrated COVID-19 and TB screening from the perspective of healthcare providers (HCPs) at a National Referral Hospital in Kampala, Uganda.

**Design/methods:**

We conducted a cross-sectional study using in-depth interviews with 12 HCPs involved in TB activities in the outpatient and emergency departments at Kiruddu National Referral Hospital, Kampala, Uganda. We explored the HCP experiences at work in the setting of COVID-19, HCP perceived effect of COVID-19 on TB screening activities at the hospital, and perceptions about social and contextual factors that might influence the willingness of HCP to integrate screening of COVID-19 and TB. We analyzed the data using an inductive thematic approach and we denoted the emergent themes as barriers to and facilitators of COVID-19/TB integrated screening. We then mapped the themes to the Capability, Opportunity, Motivation, and Behavior (COM-B) model.

**Results:**

The facilitators to integrated COVID-19 and TB screening included the availability of TB focal persons and already existing training forums at the hospital that could be utilized to strengthen the capacity of HCP to integrate COVID-19 and TB screening. The barriers included HCP’s inadequate knowledge on how to integrate screening of COVID-19 and TB, the absence of simple easy-to-use standard operating procedures and data collection tools for integrated screening, inconsistent supply of personal protective equipment (PPE), understaffing, and fear of contracting COVID-19 infection. The identified intervention functions to address the facilitators or barriers included education, persuasion, enablement, and training.

**Conclusions:**

These findings provided a basis for designing contextually appropriate interventions targeting factors that are likely to influence HCP decisions and willingness to conduct TB screening in the context of COVID-19. Future studies should evaluate the effect of addressing these barriers to the integration of COVID-19 and TB as well as the effect of this on TB case finding in high-burden TB settings.

**Supplementary Information:**

The online version contains supplementary material available at 10.1186/s43058-023-00391-w.

Contributions to the literature

- We utilized the widely used and validated COM-B model to assess determinants of integrated COVID-19 and TB screening at a national referral hospital.
- To our knowledge, this is the first qualitative study to explore barriers and facilitators to the integrated screening of COVID-19 and TB using the COM-B model.
- The barriers and facilitators identified provide a basis for developing stakeholder-informed and contextually appropriate interventions targeting factors that are likely to influence HCP decisions and willingness to conduct TB screening in the context of COVID-19.

## Background

Coronavirus disease 2019 (COVID-19) is caused by the severe acute respiratory syndrome coronavirus 2 (SARS-CoV-2) infection and is a disease of global concern since early 2020. In March 2020, the World Health Organization (WHO) declared COVID-19 a global pandemic [1]. Since then, the global and local response to COVID-19 has caused severe disruptions to the service delivery for other diseases including tuberculosis (TB) [2–6]. The commonest symptoms of TB (fever and cough) are similar to those exhibited by COVID-19 patients [7], which may negatively affect people’s health-seeking behavior for fear of stigmatization [8, 9]. This may also make healthcare providers less receptive to patients presenting with COVID-19-like symptoms from other causes such as TB [10]. Furthermore, there is an increased prevalence and a higher fatality risk for TB/COVID-19 co-infection than with patients infected with COVID-19 alone [11].

**Fig. 1:**
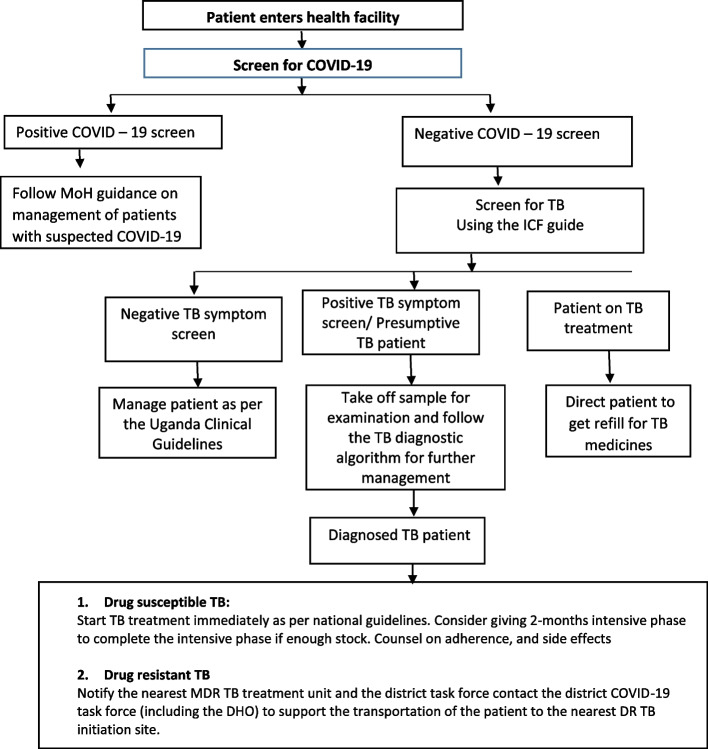
Health facility tuberculosis (TB) management plan in the context of COVID-19 guidance by Uganda Ministry of Health (MoH)

Following the COVID-19 outbreak, as has been documented elsewhere [5, 6, 9], Uganda experienced a 40% drop in TB screening by June 2020, based on the unpublished Ministry of Health (MOH) programmatic data from the District Health information system version 2 (DHIS2) [12]. In response to this very significant drop in screening for TB, the National TB and Leprosy Program (NTLP) at the Uganda MoH developed a health facility TB management plan in the context of COVID-19. The plan included the development of an algorithm to integrate screening for COVID-19 and TB (COVID-19/TB screening algorithm) (Fig. 1). Using this algorithm, all patients who present to the health facility should be screened for COVID-19. For patients who screen positive for COVID-19, the MoH guidance on the management of COVID-19 suspects should be followed. Patients who screen negative for COVID-19 should subsequently undergo TB symptom screening using the TB intensified case finding guide. Patients with a positive TB symptom screen (presumptive TB cases) should be followed by confirmatory testing with Gene Xpert MTB/RIF (Xpert; Cepheid, USA).

This qualitative study aimed to identify facilitators of and barriers to integrated COVID-19 and TB screening from the perspective of an HCP at a referral hospital in Kampala, Uganda, and map these onto the Capability, Opportunity, and Motivation model of Behavior (COM-B) and Behavior Change Wheel (BCW) framework to identify suitable intervention functions. We utilized the COM-B model to explore facilitators of and barriers to integrating COVID-19 and TB screening in behavioral terms. We chose the COM-B model, which forms part of the Behavior Change Wheel framework, to understand the capabilities, opportunities, and motivations for HCP to integrate screening of COVID-19 and TB.

**Fig. 2:**
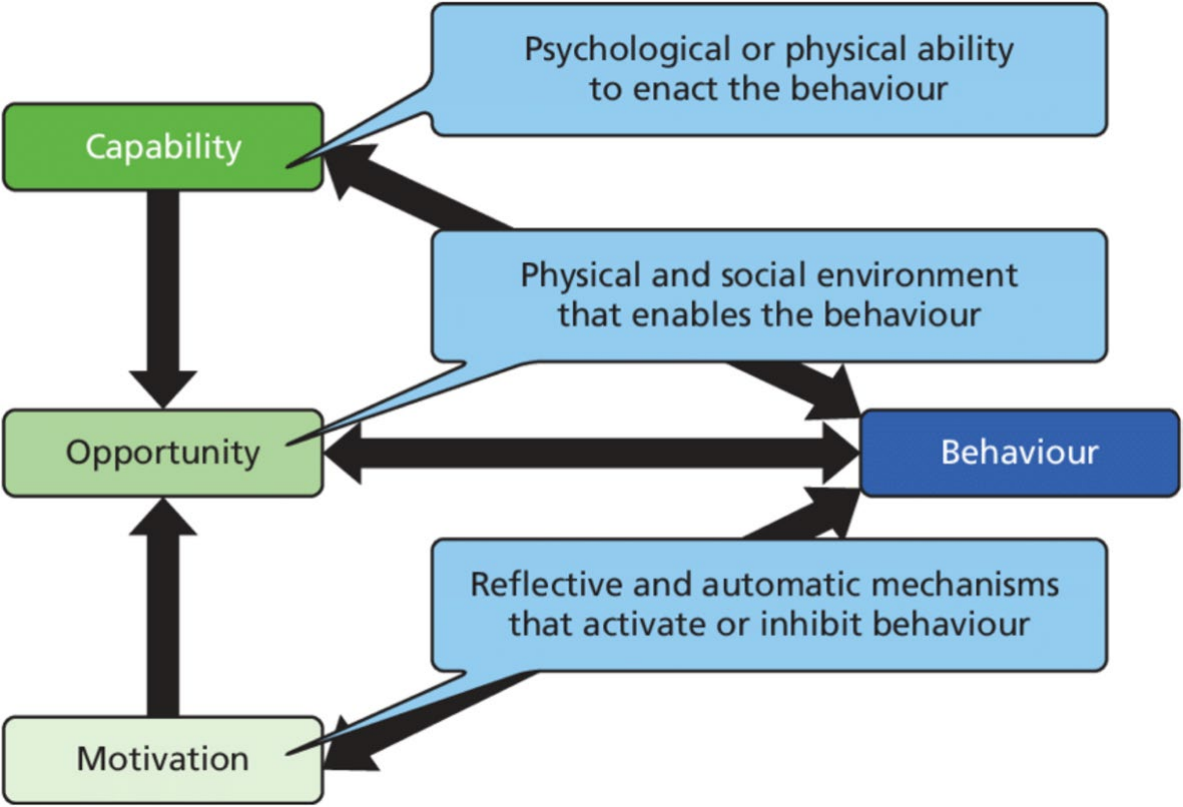
The COM-B model of behavior. Adapted from the original figure (Michie et al.)

The central principle for the COM-B is that changing any behavior requires changing capability, opportunity, and/or motivation to perform that behavior [12]. Thus, the COM-B model (Fig. 2) provides a coherent basis for exploring barriers to and facilitators [13]. This behavioral analysis based on the COM-B would inform the necessary interventions that need to be implemented to promote integrated screening of COVID-19 and TB among HCP. Furthermore, the COM-B model is key in understanding influences on individual behavior and in our case the HCPs who are the frontline stakeholders in conducting integrated screening of COVID-19 and TB.

The goal was to inform the design of a contextually appropriate strategy to integrate screening of COVID-19 and TB in Uganda.

## Methods

### Study design and setting

We conducted in-depth interviews between January 2021 and March 2021 with HCP involved in TB-related care activities (TB screening and treatment) at Kiruddu National Referral Hospital (KNRH) in Kampala, Uganda. KNRH is a 200-bed capacity hospital in Makindye Division, one of the five administrative units of Kampala, the Capital City of Uganda. The hospital specializes in Internal Medicine, burns, and plastic surgery and provides up to 500 outpatient consultations daily.

We used the Consolidated Criteria for Reporting Qualitative Research (COREQ) guidelines in reporting this qualitative study [13] (see Additional file 1). The study received ethical approvals from The AIDS support organization (TASO) Research and Ethics Committee (TASO REC 082/2020-UG-REC-009) and the Uganda National Council of Science and Technology (HS1152ES).

### Study participants and sampling

We purposively sampled HCP involved in TB-related care activities in the outpatient and emergency departments at KNRH. The maximum sample of HCP was determined by data saturation as proposed by Lincoln and Guba [14]. Lincoln and Guba propose a naturalistic inquiry as an alternative to traditional positivistic inquiry. This naturalistic inquiry is characterized by research in natural settings, purposive sampling, and inductive analysis.

We recruited different cadres of eligible HCP including medical officers, nurses, and TB community linkage facilitators. While observing the COVID-19 prevention guidelines, we approached HCP face to face as a group and we informed them that the goal of the study was to explore their perspectives that would inform the design of a contextually appropriate strategy to integrate screening for COVID-19 and TB.

### Study instruments and data collection

We developed interview guides with open-ended questions, designed to explore barriers to and facilitators of integrated COVID-19 and TB screening as perceived by HCP involved in TB-related care activities at KNRH. These included questions about HCP work experience in the setting of COVID-19 (i.e., how COVID-19 activities had affected routine TB screening) and HCP perspectives regarding the integration of COVID-19 and TB screening (i.e., their thoughts on its importance, how it could be achieved at their departments, factors that could influence their decisions to accept it or not, concerns and likely challenges to the acceptability, feasibility, and scaling up the use of the COVID-19/TB screening algorithm).

The interview guide was drafted in English, piloted, and refined using a convenience sample of HCP at the hospital who were not participating in the study. Interviews with HCP lasted between 25 and 40 min. Informed verbal consent was obtained from all participants. All interviews including the informed verbal consent were audio-recorded and transcribed verbatim.

All transcripts were de-identified and stored in a secure digital folder accessible only by the research team.

### Research team

All interviews were conducted at the Kiruddu National Referral Hospital, a setting familiar to study participants, by the local study team. The team comprised social scientists, implementation scientists, physicians, national TB policy implementers, and other health scientists. A Masters-trained social scientist (DK, male) trained the team before the data collection and supervised the data collection process. A bachelor’s trained social scientist (MM, female) conducted the initial interviews with healthcare providers while the study nurse (JN, female) attended the sessions to take notes. The social scientist and the study nurse conducted subsequent interviews with healthcare providers. The interviewers did not know the study participants before the study commencement.

### Data analysis

Four members of the research team (FCS, RK, DK, and DO) analyzed the data using a thematic approach. We preferred thematic analysis because it is suitable for examining the perspectives of different research participants, highlighting similarities and differences, and generating unanticipated insights [15]. We adopted an inductive approach using open coding that facilitated the identification of themes within the data. Initially, two analysts (RK, DK) read three similar transcripts independently familiarizing themselves with the data and documenting thoughts on potential codes and themes. Thereafter, the transcriptions were exported to Atlas. ti version 8. The team (FCS, RK, DK, and DO) then met to debrief and compare the initial codes generated by each analyst. The team discussed coding discrepancies and resolved them among themselves. We developed a coding framework and applied it to the remaining transcripts. We noted the themes emerging during the coding processes and reviewed them in subsequent team meetings where we discussed and developed a consensus on the themes documented.

We categorized the emergent themes as either potential facilitators or barriers to integrating COVID-19 and TB screening. Themes that positively influenced integrating COVID-19 and TB screening were denoted facilitators, and those that negatively influenced integrating COVID-19 and TB screening were denoted barriers. We extracted specific quotations from the transcripts to illustrate verbatim expressions of matters that appeared important.

We developed a behavioral diagnosis by mapping the emergent facilitators and barriers onto their associated COM-B domains. We then used the Behavioral Change Wheel (BCW) framework to identify potential interventions to promote the facilitators of and overcome the barriers to integrating COVID-19 and TB screening.

## Results

### Demographic characteristics of study participants

Twelve healthcare providers participated in the interviews. They included seven medical doctors, three nurses, and two community linkage facilitators. Six healthcare providers (50%) were female. The duration in service at the current post ranged from 3 months to 8 years (median 2.25 years; IQR: 1.5–4 years).

### Healthcare provider-reported facilitators of integrating COVID-19 and TB screening

Most healthcare providers reported that TB focal persons (health workers assigned to coordinate all TB-related activities at the health facility) [16] were available to support HCP to provide integrated screening for COVID-19 and TB. The presence of TB focal persons plays a facilitative role in the integration of screening for COVID-19 and TB through organizing, mentoring, encouraging, and influencing other HCP on the need for integrated COVID-19 and TB screening.

… Just like we have the TB focal persons that are already in existence. We have focal persons at the facility level, sub-health district, district, etc. The focal persons will support HCP to provide integrated screening for TB and COVID-19. (Medical Doctor at the hospital)

Healthcare providers also suggested that utilizing the already existing training forums at the health facility would strengthen the capacity of HCP to integrate COVID-19 and TB screening.

…... you will have to use existing workshops or mentorships and training for all the health workers to integrate COVID-19 and TB screening. (Medical Doctor at the hospital)

### Healthcare provider-reported barriers to integrating COVID-19 and TB screening

Most HCP reported having knowledge gaps on how to screen for TB in the context of COVID-19. As a result of these knowledge gaps, HCPs could not easily differentiate TB and COVID-19 symptoms during screening due to the overlapping symptoms in both diseases.

My experience in screening TB in the presence of COVID-19 is that sometimes you may not be sure whether you are dealing with TB or COVID-19. Because the presentations are a bit similar…but where we have some doubts, we have been referring those clients for COVID-19 testing. (Nurse at the hospital).

The knowledge gaps may also have contributed to HCP’s tendency to put more emphasis on screening for COVID-19 than TB.

….COVID-19 has masked the TB. People have now put much emphasis on COVID-19 leaving out TB. So we have seen some decline in the cases of TB cases. But it does not mean that these cases have gone down. (Medical Doctor at the hospital)

Although there was an existing TB screening tool and a recently developed COVID-19 screening algorithm, HCPs expressed a need for simple, easy-to-use standard operating procedures and data collection tools to integrate screening of COVID-19 and TB. The absence of a simple-to-use tool for integrated screening for COVID-19 and TB made it difficult to implement integrated screening.

If you have a very tedious tool for screening, they (HCP) may not do it because it consumes a lot of time. But if it is a simplified tool then it can be well utilized, it is easier to use. (Medical Doctor at the hospital)

The HCP also reported an inconsistent supply of personal protective equipment (PPE) as a constraint in integrating COVID-19 and TB screening. This caused HCPs to be less willing to screen patients for TB due to fear of contracting COVID-19 infection in the process.

If the government can equip the hospital with supplies like sanitizers, temperature guns, PPE. I think it would help health workers accept because they will know that at least our health is well protected (Nurse at the hospital)

The HCP also reported inadequate staffing levels, coupled with very busy outpatient and emergency departments at the hospital as a hindrance to the integrated screening of COVID-19 and TB. This is because the HCP perceived integrated screening for TB and COVID-19 as an additional workload to the already overworked staff.

One of the concerns is the heavy workload because at the end of the day… You find that it’s one person who is at the unit to do the screening of several patients. (Medical Doctor at the hospital)

We are still understaffed in most places because you have two nurses treating patients on the whole floor or level and yet they want to remove one nurse and take her somewhere else. (Medical Doctor at the hospital)

Even where there was an adequate supply of PPE, some HCPs were afraid of contracting COVID-19 infection during integrated screening for COVID-19 and TB due to a concern that integrated screening would increase their time of exposure to COVID-19 infection.

Anything to do with COVID-19, I don’t want to know, even the ones we are working with [health workers], if she reads a file and sees the word COVID-19 anywhere, that patient may not be seen and may not or delay to receive treatment…. (Nurse at the hospital)

Some healthcare providers also raised the concern of unclear compensation for health workers who contract COVID-19 while on duty. They added that their safety is a concern because they are likely to be infected with COVID-19 yet they will not be compensated. This is likely to demotivate HCP to conduct integrated screening of COVID-19 and TB.

Of course, people need allowances, without allowances they are not going to work [screen for COVID-19 and TB], actually for us we don’t have COVID-19 allowances at this hospital, because they say we don’t treat COVID-19 because it is in the communities, yet we treat COVID-19 here. Screening for both COVID-19 and TB is okay… (Medical Doctor at the hospital).

Healthcare provider-reported barriers to and facilitators of integrating COVID-19 and TB screening are summarized in Table 1.

**Table 1:** Healthcare provider-reported facilitators of and barriers to integrated COVID-19 and TB screening at Kiruddu National Referral Hospital, Kampala, Uganda

Potential facilitators	Potential barriers
**TB focal persons are available to support healthcare providers to provide integrated screening for TB and COVID-19** “… Just like we have the TB focal persons that are already in existence. We have focal persons at the facility level, sub-health district, district, etc. The focal persons will support HCP to provide integrated screening for TB and COVID-19.” **(Medical Officer at the hospital) (Nurse at the hospital)**	**Healthcare providers lacked adequate knowledge on how to integrate screening of TB and COVID-19** “My experience in screening TB in the presence of COVID-19 is that sometimes you may not be sure whether you are dealing with TB or COVID-19. Because the presentations are a bit similar…but where we have some doubts, we have been referring those clients for COVID-19 testing.” **(Nurse at the hospital)**
**Healthcare providers also suggested that utilizing the already existing training forums at the health facility would strengthen the capacity of HCP to integrate COVID-19 and TB screening.** “…... you will have to use existing workshops or mentorships and training for all the health workers to integrate COVID-19 and TB screening.” **(Medical Doctor at the hospital)**	**Lack of simple standard operating procedures for integrated screening of TB and COVID-19** “If you have a very tedious tool for screening, they (HCP) may not do it because it consumes a lot of time. But if it is a simplified tool then it can be well utilized, it is easier to use.” **(Medical Officer at the hospital)**
	**Lack of consistent supply of personal protective equipment (PPE)** “If the government can equip the hospital with equipment like sanitizers, temperature guns, PPE. I think it would help health workers accept because they will know that at least our health is well protected **(Nurse at the hospital)**
	**Understaffing at the outpatient and emergency departments** “We are still understaffed in most places because you have two nurses treating patients on the whole floor or level and yet they want to remove one nurse and take her somewhere else.” **(Medical Officer at the hospital)**
	**Healthcare providers fear contracting COVID-19 infection during integrated screening** “Anything to do with COVID-19, I don’t want to know, even the ones we are working with [health workers], if she reads a file and sees the word COVID-19 anywhere, that patient may not be seen and may not or delay to receive treatment”. **(Nurse at the hospital)**
	**Lack of risk allowance for healthcare providers to conduct integrated screening of TB and COVID-19** “..Of course, people need allowances, without allowances they are not going to …actually for us we do not have COVID allowances at this hospital, because they say we don’t treat COVID because COVID is in the communities yet we treat COVID here. That one of the factors that limit health workers involved in screening for COVID, screening for both can be there so no problem but COVID mostly health workers are not interested.” **(Medical Officer at the hospital)**

*Abbreviations*: *HCP* healthcare providers, *TB* tuberculosis, *PPE* personal protective equipment

### Behavioral diagnosis and intervention functions

We categorized the reported barriers and facilitators of integrated COVID-19 and TB screening within the domains of the COM-B model to obtain the behavioral diagnosis. For example, identified barriers including lack of simple standard operating procedures for integrated screening of TB and COVID-19, inconsistent supply of PPE, and understaffing at the outpatient and emergency departments were mapped to the physical opportunity construct of the COM-B model. Table 2 summarizes the HCP-reported facilitators and barriers expressed in terms of their behavioral determinants within the COM-B model.

**Table 2:** Barriers to and facilitators for integrated COVID-19 and TB screening at Kiruddu National Referral Hospital mapped to the COM-B model

COM-B constructs	Barriers	Facilitators
**Psychological capability**	Healthcare providers lacked adequate knowledge on how to integrate screening for COVID-19 and TB	
**Physical capability**		
**Physical opportunity**	Lack of simple standard operating procedures for integrated screening of COVID-19 and TB	
	Inconsistent supply of personal protective equipment (PPE)	
	Understaffing at the outpatient and emergency departments	
	Lack of data collection tools and databases for integrated screening of COVID-19 and TB	
**Social opportunity**		TB focal persons are available to support healthcare providers to provide integrated screening for COVID-19 and TB
**Reflective motivation**	Healthcare providers fear contracting COVID-19 infection during integrated screening	Already existing training forums at the health facility could be utilized to strengthen the capacity of HCP to integrate COVID-19 and TB screening
**Automatic motivation**	Lack of risk allowance for healthcare providers to conduct integrated screening of COVID-19 and TB	

*Abbreviations*: *HCP* healthcare provider, *TB* tuberculosis, *PPE* personal protective equipment

We linked the behavioral diagnosis obtained using the COM-B model (Table 2) to the Behavioral Change Wheel (BCW) framework and identified appropriate potential intervention functions that could serve to address the reported barriers and facilitators and thereby enhance the acceptance of integrating screening of COVID-19 and TB. These are summarized in Tables 3 and 4. For example, HCP reported inadequate knowledge on how to integrate screening for COVID-19 and TB. As summarized in Table 5 using *education and training* as intervention functions, HCP can be equipped with the necessary knowledge and skills through training sessions. Similarly, using *enablement* as an intervention function, HCP can be provided simplified standard operating procedures for integrated screening of COVID-19 and TB, provided an adequate supply of PPE, and improve the staffing levels in these departments as enablers to facilitate integrated screening for COVID-19 and TB.

**Table 3:** Summary of identified facilitators and linked intervention functions

Capability		Opportunity	Motivation	Intervention functions
Psychological	Physical	Social	Reflective	
		Availability of TB focal persons are available to support HCP	Already existing training forums at the health facility could be utilized to strengthen the capacity of HCP to integrate COVID-19 and TB screening	
				Education
		**X**	**X**	Persuasion
		**X**	**X**	Enablement
				Training

*Abbreviations*: *HCP* healthcare provider, *TB* tuberculosis

**Table 4:** Summary of identified barriers and linked intervention functions

Capability	Opportunity				Motivation		Intervention functions
Psychological	Physical				Reflective	Automatic	
Inadequate knowledge to integrate screening of TB and COVID-19	Lack of SOPs	Lack of consistent supply of PPE	Understaffing	Lack of data collection tools	Fear of contracting COVID-19 infection	Lack of risk allowance	
**X**					**X**		Education
					**X**	**X**	Persuasion
	**X**	**X**	**X**	**X**		**X**	Enablement
**X**							Training

*Abbreviations*: *PPE* personal protective equipment, *SOPs* standard operating procedures, *TB* tuberculosis

**Table 5:** Summary of potential strategies for each intervention function to address identified barriers to integrated COVID-19 and TB screening at Kiruddu National Referral Hospital

Intervention function	Potential strategy
Education	a) Provide the HCP with the necessary knowledge
Training	a) Conduct training sessions for skills acquisition
Persuasion	a) Improve infection prevention practices b) Conduct performance reviews
Enablement	a) Provide simplified SOPs and data collection tools b) Ensure a consistent supply of PPE c) Improve the staffing levels, e.g., task shifting

*Abbreviations*: *PPE* personal protective equipment, *SOPs* standard operating procedures

## Discussion

In this formative cross-sectional study, we explored HCP work experience in the setting of COVID-19, the perceived effect of COVID-19 on TB screening, and perceptions about social and contextual factors that might influence their willingness to screen for both diseases at Kiruddu National Referral Hospital, Kampala, Uganda. We utilized the COM-B model to explore barriers to and facilitators of integrating screening for COVID-19 and TB [17].

We found that COVID-19 was a real threat to the provision of TB services since HCP at this large hospital were not very well prepared to integrate COVID-19 and TB services at the time. The key barriers to integrating COVID-19 and TB screening included a lack of simple standard operating procedures for integrated screening of COVID-19 and TB, inconsistent supply of PPE, understaffing, and HCPs’ fear of contracting COVID-19 infection. The key facilitators for integrating COVID-19 and TB screening included the availability of TB focal persons to support HCP to provide integrated screening for COVID-19 and TB and already existing training forums at the hospital. These would be utilized to strengthen the capacity of HCP to integrate screening for both COVID-19 and TB.

Many of the HCP also reported a lack of simple-to-use tools such as standard operating procedures, screening algorithms, and data collection tools to support integrated screening of COVID-19 and TB. Simple tools in the form of protocols have been shown to facilitate the integration of different programs in clinical settings [18, 19].

Generally, the HCP reported a lack of consistent supply of PPE, and most of them feared contracting the COVID-19 infection as hindrances to the provision of integrated screening for COVID-19 and TB. These fears expressed by the HCPs are consistent with what has been reported from studies in other settings [20–22]. Two studies have previously affirmed that appropriate provision of PPE, training in its appropriate use, and comprehensive and consistent guidance is fundamental in reducing the fear of contracting COVID-19 among HCPs [23, 24]. Some HCPs were not willing to evaluate patients suspected to have COVID-19 for TB. This, however, may not have huge negative implications on the wider care of patients with COVID-19 since it was expressed by only two out of the 12 HCPs interviewed for the study.

Our findings add to the growing evidence showing that countries and communities need to design contextually appropriate and stakeholder-informed strategies that adapt active case finding for TB during the COVID-19 pandemic for the continuity of TB services [25–29]. In a summary of three operation research studies conducted in the capital cities of three African countries (Kenya, Malawi, and Zimbabwe), Harries et al. assessed whether real-time monthly surveillance of TB and HIV activities compared with quarterly surveillance would minimize the anticipated negative impact of COVID-19 pandemic on TB and HIV services [30]. The three studies showed a decline of 31.2%, 40.6%, and 45.6% in the numbers of people presenting with presumptive pulmonary TB for investigation in the three respective countries for the two periods (March 2020–February 2021 compared to the immediate pre-COVID-19 period of March 2019–February 2020).

Following the institution of measures (integrated screening and fast-tracking of investigations for COVID-19 and TB in patients presenting with respiratory symptoms; active TB case finding in hot spots in the city and improved contact tracing in selected facilities) to improve TB case detection, there was only a 5% increase in the numbers of people presenting with presumptive pulmonary TB for investigation in Kenya. On the other hand, the numbers in Malawi and Zimbabwe remained far below the baseline period. However, these studies do not describe the steps taken to understand the perspectives of key stakeholders such as HCP in this setting and this might have contributed to suboptimal improvement in the target outcomes.

We utilized the identified facilitators for and barriers to the integrated screening of COVID-19 and TB, and the BCW intervention functions to develop strategies that can be easily implemented. We trained 37 HCPs on the COVID19-TB algorithm through a series of short interactive sessions to close the knowledge and skills gap [31]; we printed and distributed job aids of the COVID-19/TB algorithm and simple-to-use data collection tools at all the screening points to overcome the absence of guidelines on how to screen for TB in the context of COVID-19 and absence of tools for data collection, to promote integrated screening. Finally, we procured and distributed PPE to the HCP to overcome the lack of consistent supply of PPE and the related fear of contracting COVID-19 infections [23, 24].

The strength of our study is that it utilizes implementation science approaches including stakeholder engagement and the COM-B model in this formative assessment. We engaged the HCPs who are key stakeholders to understand the contextual factors that may affect their willingness to integrate screening for COVID-19 and TB. Several implementation science studies have demonstrated that engaging key stakeholders in the development of interventions leads to buy-in from the stakeholders and fosters ownership of the intervention and the intervention is more likely to be effective when compared to interventions that are designed without the involvement of the key stakeholder input [32, 33].

By using the widely applied COM-B model, we made a behavioral diagnosis of the possible challenges to implementing integrated screening for COVID-19 and TB. The framework also provides the behavioral change techniques, which we utilized to design contextually appropriate interventions.

A potential limitation of our study is that the findings from our study are from a single urban national referral hospital and some contextual factors may not be generalizable in different settings. However, many such centers in Uganda and other sub-Saharan African countries often have similar challenges [34, 35].

Another limitation of this study is the inability of us to explain the potential influence of organizational-level behavior on HCPs’ behavior and perceptions. The use of other models like the social-ecological model appreciates that individual behavior is a multifaceted and bidirectional relationship between individuals and their environments [36, 37]. As a result, individual behavior is affected by multiple levels of influence and can be shaped by the wider social environment. This formative study, however, focused on frontline HCPs, who were directly responsible for the delivery of the service. Therefore, using the COM-B, we were able to identify the key barriers to and facilitators of implementing integrated screening for COVID-19 and TB.

Lastly, perceptions do not always match up with behaviors and the COM-B model may not explicitly explain the link between perception and HCP behavior.

## Conclusions

The findings of our study highlight the barriers to and facilitators for integrated COVID-19 and TB screening. These findings provide a key stakeholder-informed basis for designing contextually appropriate interventions targeting factors that are likely to influence HCP decisions and willingness to accept the use of this algorithm for integrated COVID-19 and TB screening in similar settings. Future studies should evaluate the effect of addressing these barriers to the integration of COVID-19 and TB as well as the effect of this on TB case finding in high-burden TB settings.

## Supplementary Information

**Additional file 1.** Consolidated Criteria for Reporting Qualitative Research (COREQ): 32-item checklist.

## Data Availability

The datasets used and/or analyzed during the current study are available from the corresponding author upon reasonable request.

## Abbreviations

BCW: Behavior Change Wheel framework
COM-B: Capability, Opportunity, Motivation, and Behavior
COVID-19: Coronavirus disease 2019
HCP: Healthcare provider
KNRH: Kiruddu National Referral Hospital
MoH: Ministry of Health
NTLP: National TB and Leprosy Program
PPE: Personal protective equipment
TB: Tuberculosis
WHO: World Health Organization
